# Supplementary material for: Effect of Novel Gastro-Duodenal Flow Restrictor on Relative Weight Loss and Glucose Levels in a Porcine Model: A Pilot Randomized Study
Source: Nutrients. 2022 Jun 21;14(13):2563. doi: 10.3390/nu14132563 (PMC9268118; doi:10.3390/nu14132563)
Supplement: Supplementary file 1 [file nutrients-14-02563-s001.zip › Supplementary legends-revised.pdf]

Supplementary Legends

Supplementary Figure S1. Line graphs showing the percentages of relative weight loss in the control group compared to case porcine groups (placement of G-DFR for 28 days = 1, placement of G-DFR for 42 days = 1, placement of G-DFR for 60 days = 1, and placement of G-DFR for 90 days = 1). The relative weight loss in the control group compared to the case group on days 70 (98.9 vs. 42.1, $p = 0.013$) and 9 0 (140.6 vs. 85.2, $p = 0.017$) was significantly different. G-DFR, Gastro-duodenal flow restrictor.

Supplementary Figure S2. Bar graphs representing the differences in ghrelin hormone levels between the control and case groups (60 days of G-DFR placement = 1 and 90 days of G-DFR placement = 1) by observation time. The ghrelin hormone level was significantly different between the control and case groups on days three (p= 0.030) and 28 (p= 0.012) after the placement of G-DFR. G-DFR, Gastro-duode nal flow restrictor.

Supplementary Figure S3. Biochemical examination results showing alanine aminotransferase (left) a nd cholesterol (right) levels in the porcine study (three in control group and two in case group [60 day s of G-DFR placement = 1, 90 days of G-DFR placement = 1]). G-DFR, Gastro-duodenal flow restrictor.

Supplementary Figure S4. Endoscopic evaluation for porcine stomach (left) and proximal duodenum (right) following distal migration of G-DFR.

Supplementary Figure S5. A woven metallic wire of G-DFR before coating with PTFE of measured le ngth.

Supplementary Video S1. When the flow of gastroduodenal fluid in G-DFR was evaluated, injected contrast slowly ran through distal PTFE skirt of G-DFR after placement of G-DFR
