# Supplementary figures and images for: Effect of Novel Gastro-Duodenal Flow Restrictor on Relative Weight Loss and Glucose Levels in a Porcine Model: A Pilot Randomized Study
Source: Nutrients. 2022 Jun 21;14(13):2563. doi: 10.3390/nu14132563 (PMC9268118; doi:10.3390/nu14132563)

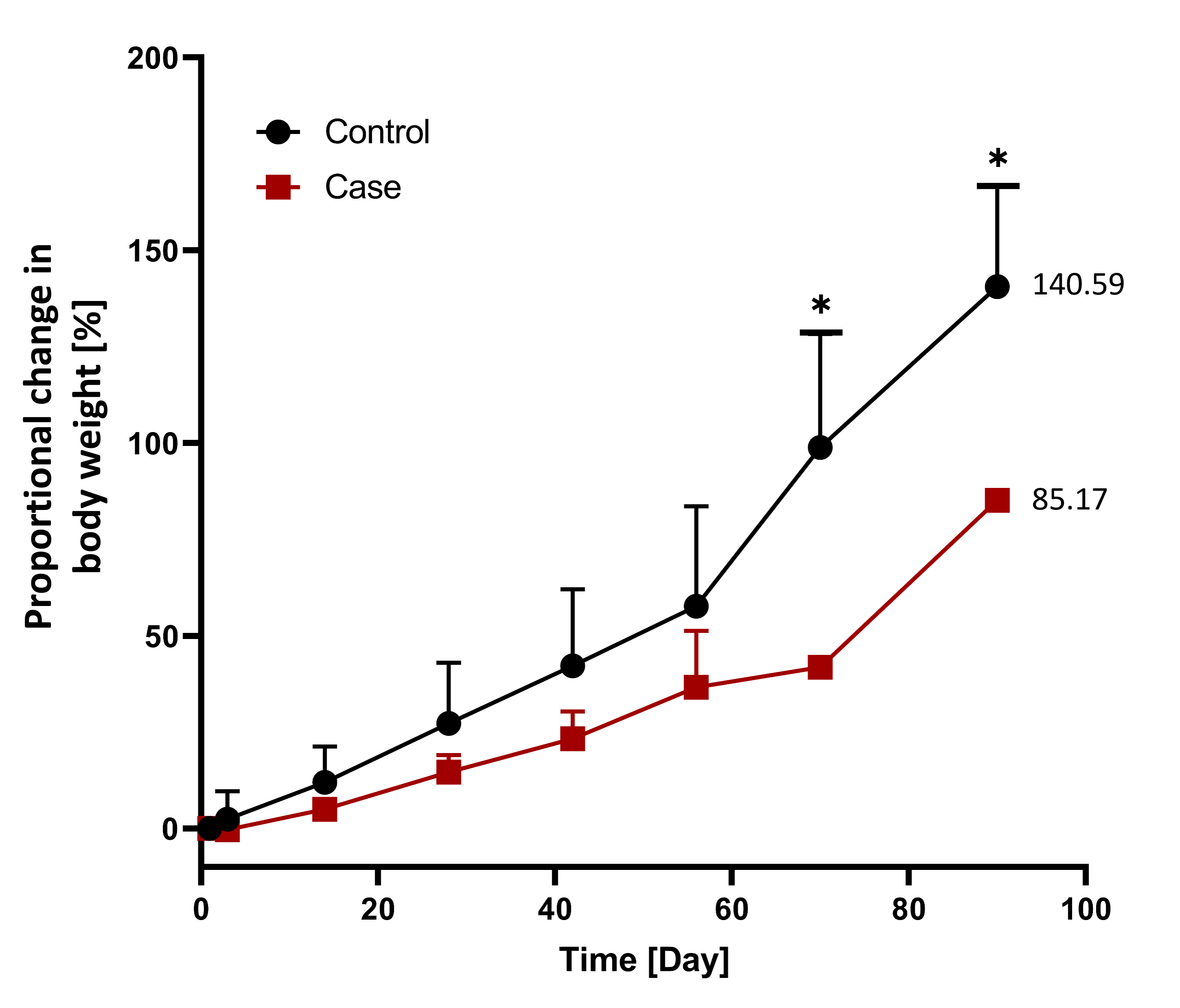

Supplement: Supplementary file 1 [file nutrients-14-02563-s001.zip › Figure S1.tif]

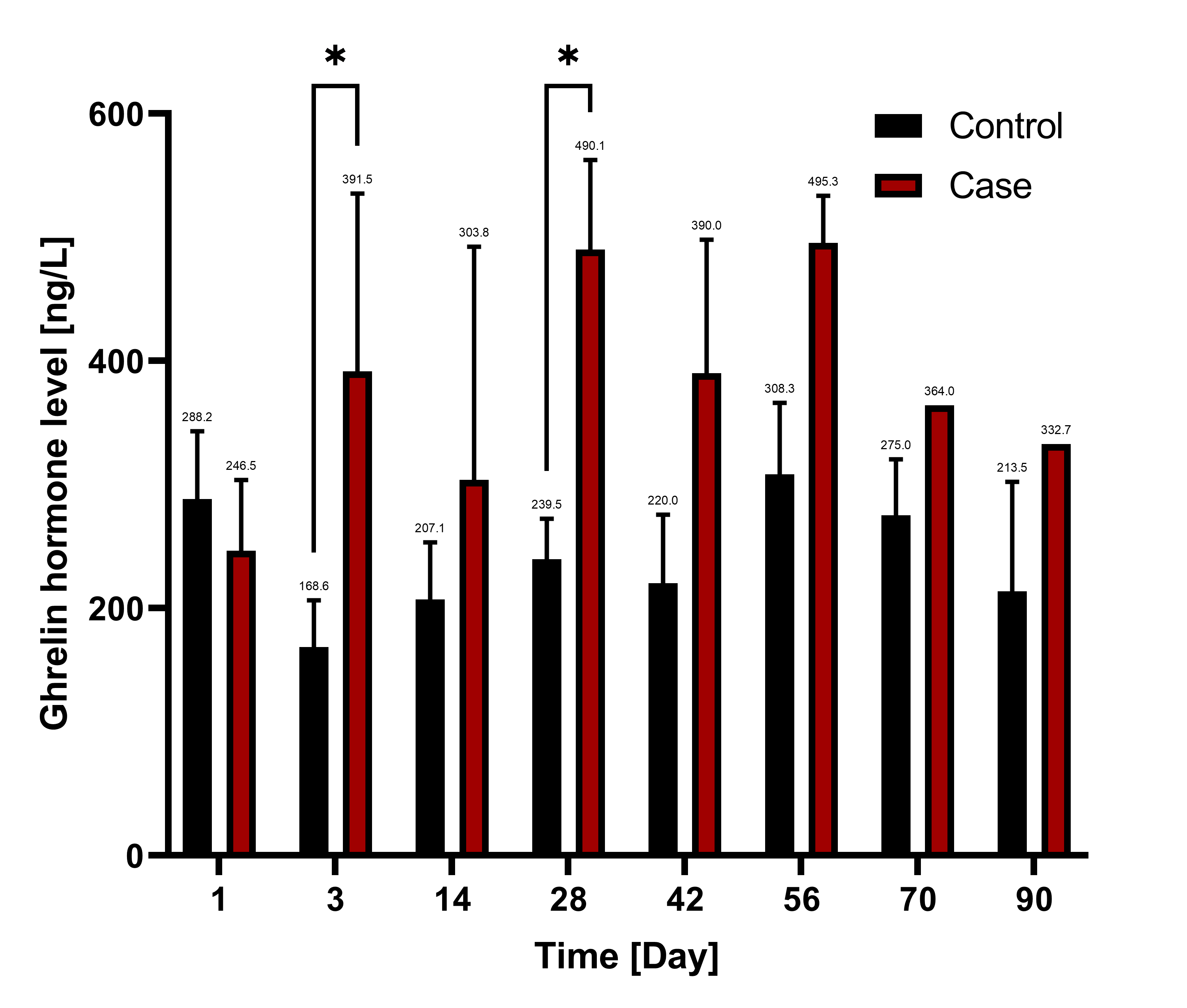

Supplement: Supplementary file 1 [file nutrients-14-02563-s001.zip › Figure S2.tif]

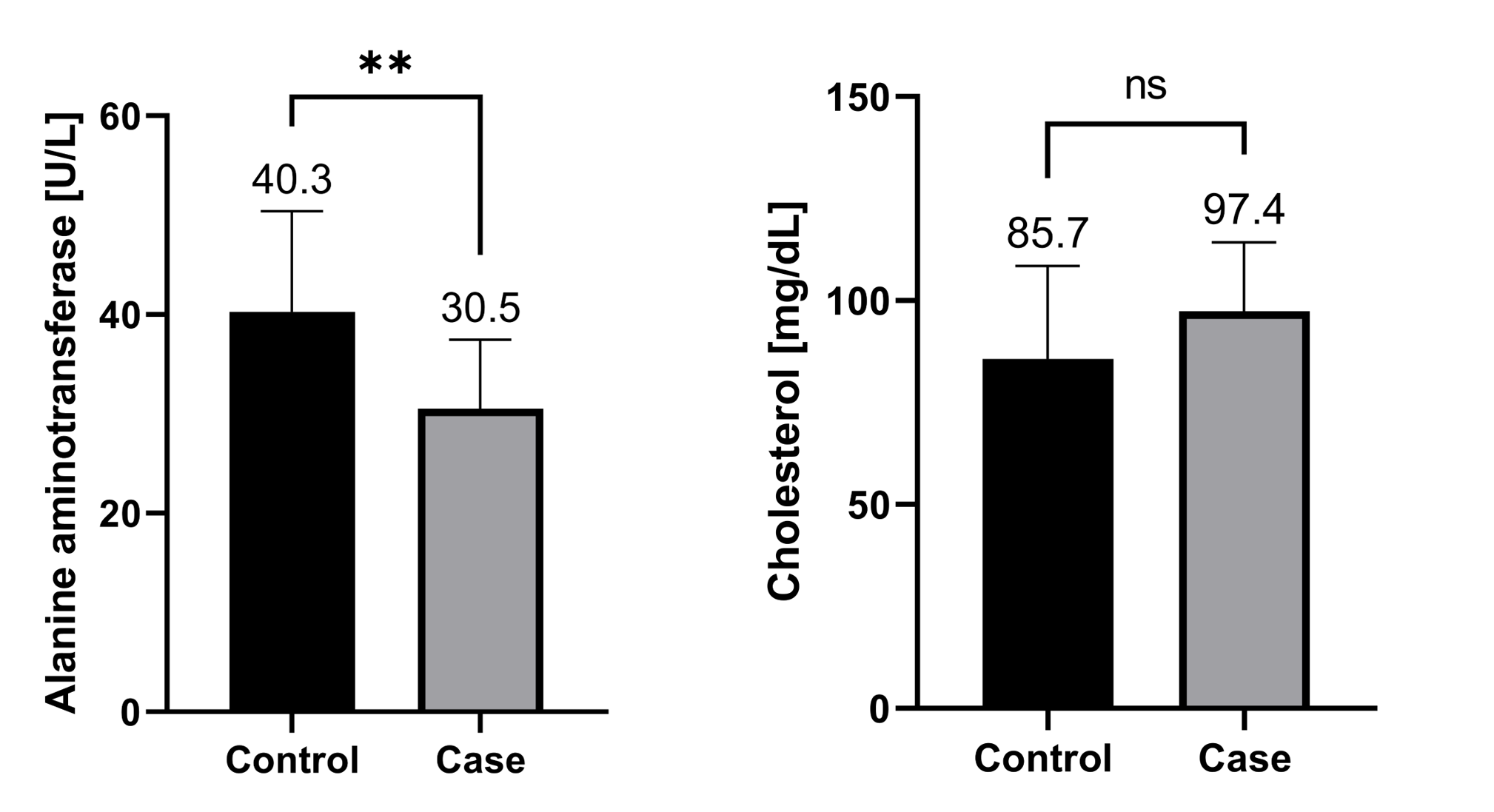

Supplement: Supplementary file 1 [file nutrients-14-02563-s001.zip › Figure S3.tif]

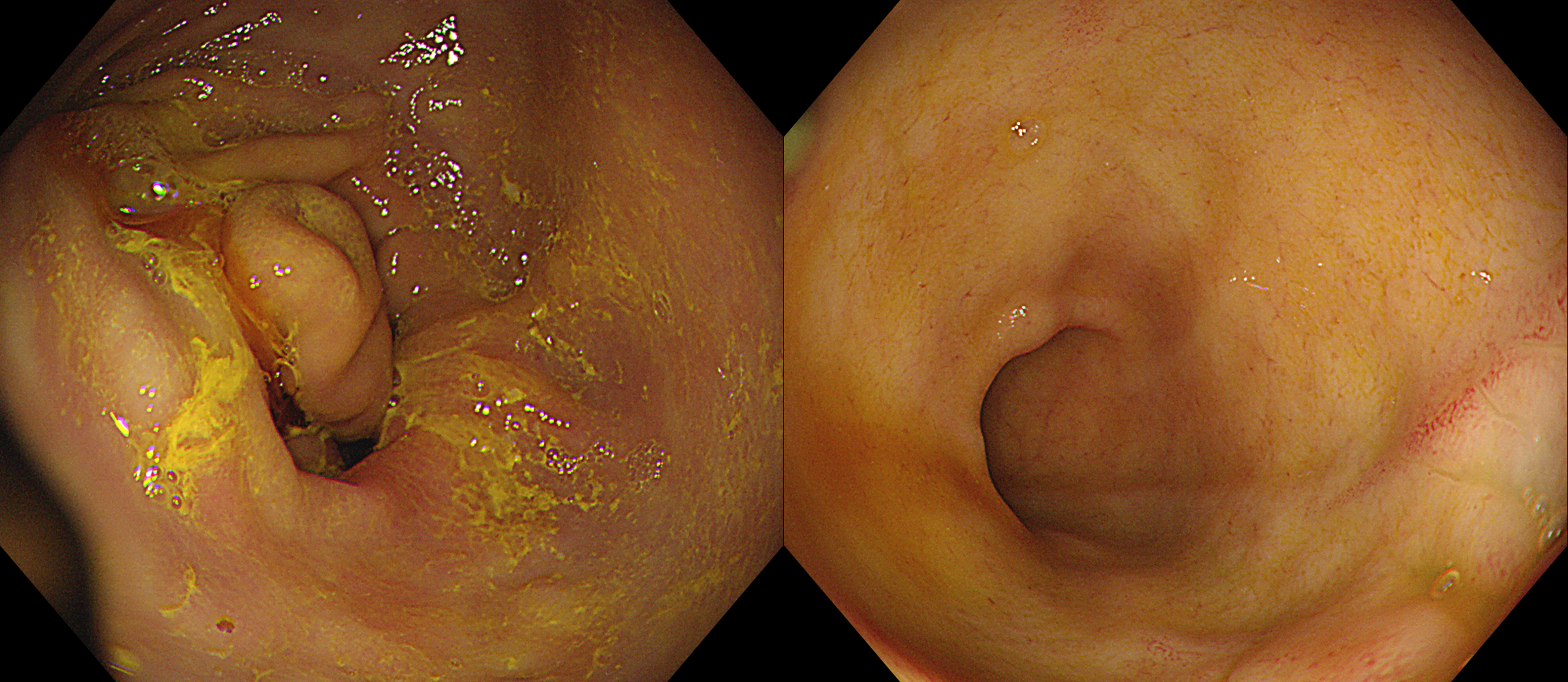

Supplement: Supplementary file 1 [file nutrients-14-02563-s001.zip › Figure S4.tif]

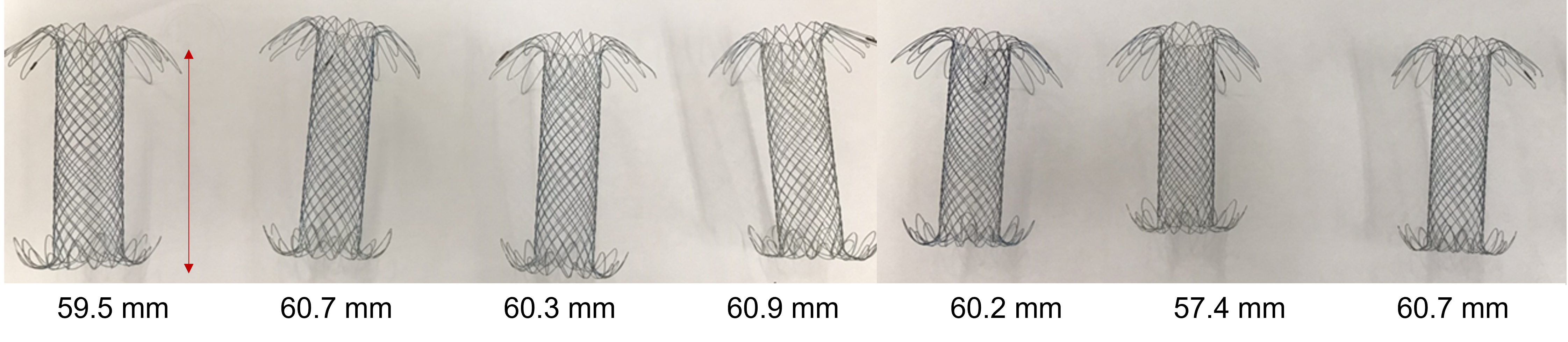

Supplement: Supplementary file 1 [file nutrients-14-02563-s001.zip › Figure S5.tif]
